# Does additional antimicrobial treatment have a better effect on URTI cough resolution than homeopathic symptomatic therapy alone? A real-life preliminary observational study in a pediatric population

**DOI:** 10.1186/s40248-015-0022-3

**Published:** 2015-08-07

**Authors:** Alessandro Zanasi, Salvatore Cazzato, Massimiliano Mazzolini, Carla Maria Sofia Ierna, Marianna Mastroroberto, Elena Nardi, Antonio Maria Morselli-Labate

**Affiliations:** Italian Association for Cough Study (AIST), Via Mazzini, 12, 40138 Bologna, Italy; Department of Pediatrics, Alma Mater Studiorum - University of Bologna, Bologna, Italy; Respiratory Medicine and Intensive Care Unit, Sant’Orsola Malpighi Hospital, Alma Mater Studiorum - University of Bologna, Bologna, Italy; Department of Medical and Surgical Sciences, Alma Mater Studiorum - University of Bologna, Bologna, Italy

**Keywords:** Anti-bacterial agents, Antitussive agents, Cough, Homeopathy, Respiratory tract infections

## Abstract

**Background:**

The effectiveness of a homeopathic syrup on cough has been demonstrated in an adult population in a previous double-blind randomized study. The present prospective observational study investigated children affected by wet acute cough caused by non-complicated URTIs, comparing those who received the homeopathic syrup *versus* those treated with the homeopathic syrup plus antibiotic.

**Objectives:**

The aims were: 1) to assess whether the addition of antibiotics to a symptomatic treatment had a role in reducing the severity and duration of acute cough in a pediatric population, as well as in improving cough resolution; 2) to verify the safety of the two treatments.

**Methods:**

Eighty-five children were enrolled in an open study: 46 children received homeopathic syrup alone for 10 days and 39 children received homeopathic syrup for 10 days plus oral antibiotic treatment (amoxicillin/clavulanate, clarithromycin, and erythromycin) for 7 days. To assess cough severity we used a subjective verbal category-descriptive (VCD) scale.

**Results:**

Cough VCD score was significantly (*P* < 0.001) reduced in both groups starting from the second day of treatment (−0.52 ± 0.66 in the homeopathic syrup group and −0.56 ± 0.55 in children receiving homeopathic syrup plus oral antibiotic treatment). No significant differences in cough severity or resolution were found between the two groups of children in any of the 28 days of the study. After the first week (day 8) cough was completely resolved in more than one-half of patients in both groups. Two children (4.3 %) reported adverse effects in the group treated with the homeopathic syrup alone, *versus* 9 children (23.1 %) in the group treated with the homeopathic syrup plus antibiotics (*P* = 0.020).

**Conclusions:**

Our data confirm that the homeopathic treatment in question has potential benefits for cough in children as well, and highlight the strong safety profile of this treatment. Additional antibiotic prescription was not associated with a greater cough reduction, and presented more adverse events than the homeopathic syrup alone.

## Background

Acute cough is a very common problem for children; the majority of them have up to five viral upper respiratory tract infections (URTIs) with cough every year [1], usually self-limiting within 3 weeks [2, 3]. Generally, such frequency of cough is more applicable to young children than to childhood and adolescence [2, 3]. Acute cough in children may last over 20 days and become worrying for the young patients and their parents [4–6]. Symptomatic treatment is often prescribed after a medical consultation, although its effectiveness it is still a matter of debate [7–9], and - contrary to recommendations - antibiotics are frequently administered to children with acute persistent cough [10–13].

A survey in the United States found that antibiotics were prescribed to 44 % of patients with common cold, to 46 % with upper respiratory tract infections and to 75 % with bronchitis. Children aged 0 to four years received 53 % of all antibiotics prescribed to the pediatric population [14].

A Cochrane review of antibiotic use for cough and common cold concluded that there was not enough evidence of important benefits in the treatment of URTI, whereas there was a significant increase in adverse effects associated with antibiotic use [15].

However, parents are rarely satisfied with in the watchful approach, and often have an expectation that antibiotics should be prescribed [16]. The aim of this preliminary study was to evaluate if the addition of antibiotics to a symptomatic treatment (homeopathic syrup) improves cough resolution in pediatric patients with acute cough due to uncomplicated URTI.

## Methods

We conducted an open prospective analysis of acute cough visits in four ambulatory settings of pediatric practitioners over a one-year period from December 2013 to December 2014. The study considered only patients affected by wet acute cough caused by non-complicated URTIs who either received a homeopathic syrup alone (Stodal® 200 mL, Boiron SA, Messimy, France) or were treated with the same homeopathic syrup plus antibiotic. The homeopathic syrup was composed of: Anemone pulsatilla 6 CH, Rumex crispus 6 CH, Bryonia dioica 3 CH, Ipecacuanha 3 CH, Spongia tosta 3 CH, Sticta pulmonaria 3 CH, Antimonium tartaricum 6 CH, Myocarde 6 CH, Coccus cacti 3 CH, Drosera MT. The effectiveness of this homeopathic syrup on cough was investigated in our previous double-blind randomized study [17]. The syrup’s dosage was 5 mL 4 times per day. Choices of antibiotic use, as well as antibiotic type and dosage, were left to the discretion of the physician for each individual patient.

### Endpoints

The first endpoint was to assess whether the addition of antibiotics to a symptomatic treatment had a role in reducing the severity and duration of acute cough in a pediatric population, as well as in improving cough resolution. The second goal was to verify the safety of the two treatments.

### Experimental design

Study assessment was carried out through an analysis of medical records (including patients’ history, clinical examination and therapy). All participants filled in a validated standardized pediatric cough diary (verbal category-descriptive scale: VCD) to grade the severity of their cough [18]. The VCD was compiled daily by the patients, assisted by their parents, for 28 consecutive days starting from the first visit. This cough-scoring diary had been previously validated against an objective cough meter measure, and changes in this subjective cough rating were shown to reflect changes in cough counts [18]. The VCD score we used consisted of 6 discrete values: 0 – no cough; 1 – one short period of mild cough without hardship; 2 – some short periods of cough without much hardship; 3 – frequent coughing that does not affect normal daily life or sleep; 4 – serious coughing that is very frequent and interferes with normal daily life or sleep; 5 – distressing continuous coughing that did not stop for 24 h. Cough was considered resolved when a score of less than 2 was reached.

Patients were re-examined at the end of the study and any adverse events were also reported.

### Patients

Eighty-five children were found eligible to be enrolled in the study. Forty-six patients received homeopathic syrup alone for 10 days (Group 1) and thirty-nine children received homeopathic syrup for 10 days plus oral antibiotic treatment (amoxicillin/clavulanate, clarithromycin, and erythromycin) for 7 days (Group 2).

The inclusion criteria were: age between 4 and 15 years, and cough induced by URTIs lasting 5 days or less. Children with pre-existing respiratory problems and/or who had antibiotic treatment or any other medication that might affect the cough symptom within 5 days were excluded from the study.

The baseline characteristics of the two studied groups are shown in Table 1. The two groups proved comparable with respect to sex, age and time from onset of cough. No significant differences in baseline severity of cough were found between male and female patients both in the overall population (*P* = 0.366) and within the two groups (*P* = 0.719 in Group 1 and *P* = 0.322 in Group 2).

**Table 1:** Characteristics of the studied children affected by wet acute cough caused by non-complicated URTI. Data are shown as frequencies or mean ± standard deviation

	Group 1: Homeopathic syrup alone (n = 46)	Group 2: Homeopathic syrup plus antibiotic treatment (n = 39)	*P* value
Gender:			0.830^a^
Male	20 (43.5 %)	18 (46.2 %)	
Female	26 (56.5 %)	21 (53.8 %)	
Age (years)	8.2 ± 2.9	8.5 ± 2.9	0.674^b^
Time from the onset of cough (days)	3.9 ± 1.0	3.8 ± 0.9	0.785^b^
Verbal category-descriptive scale at baseline (VCD)	3.96 ± 0.73	4.00 ± 0.65	0.763^b^

^a^Fisher’s exact test

^b^Kruskal-Wallis test

### Ethics

The research was promoted by the Italian Association for Cough Study (AIST) and was conducted according to the Helsinki declaration. The protocol was approved by the Institutional Review Board and the informed consent was obtained by the legal guardians of the enrolled children.

### Sample size and power analysis

To evaluate sample size, we hypothesized--for this study performed on children--the same results obtained previously in adults after 7 days of administering the same homeopathic syrup [17] (i.e., a difference between groups of VCD equal to 0.7 and a within-group standard deviation of 1.0). Based on these values, we needed to study a total of at least 76 subjects (i.e., 38 subjects in each group, hypothesizing an equal distribution of patients among the two groups) to be able to reject the null hypothesis with probability (power) equal to 0.90 at a significance level of 0.05. The sample size was estimated by means of the “PS Power and Sample Size Calculations” software (Version 3.0.43; Department of Statistics of the Vanderbilt University, Nashville, TN, USA; http://biostat.mc.vanderbilt.edu/wiki/Main/PowerSampleSize) according to the Dupont and Plummer procedure [19, 20].

### Statistical analysis

Frequencies and mean values ± standard deviation were used as descriptive statistics. The two groups of children were compared by means of the Fisher’s exact and the Kruskal-Wallis tests, while the Wilcoxon matched-pairs signed-rank test was used to test the changes of the VCD scale observed *versus* the basal values. The IBM SPSS Statistics package (Version 21; IBM Co., Armonk, NY, USA) was used to analyze the data. Two-tailed *P* values less than 0.05 were considered statistically significant.

## Results

### Cough severity

**Fig. 1:**
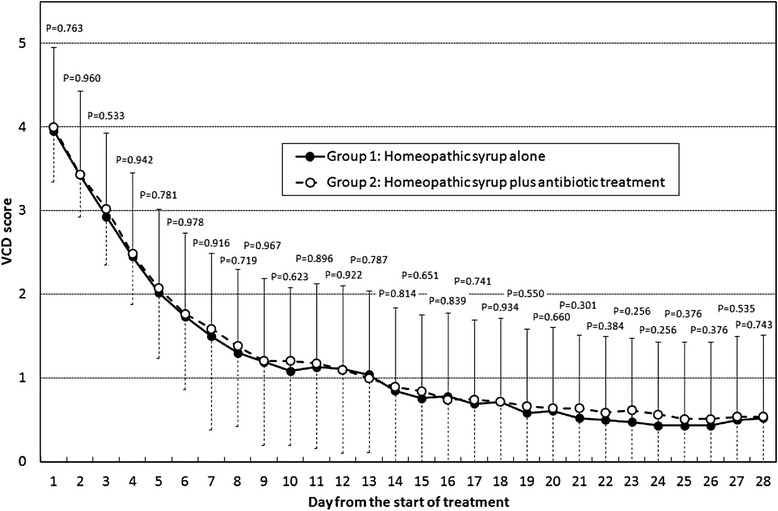
Behavior of verbal category-descriptive (VCD) scale of cough during the whole observational period in children affected by wet acute cough caused by non-complicated URTI. Data are shown as mean ± standard deviation and the Kruskal-Wallis test was applied

Figure 1 shows the behavior of the verbal category-descriptive (VCD) scale of cough during the entire observational period. Cough severity was comparable between the two groups at baseline (Day 1; *P* = 0.763) as well as on all other days of the study. We found a highly significant (*P* < 0.001) improvement in cough during the whole observational period from day 2 to day 28, both in children treated with syrup alone and in those treated with syrup plus antibiotics, with a non-significant difference in progressive reduction of cough severity between the two groups (Table 2).

**Table 2:** Improvement of cough during the whole observational period in children affected by wet acute cough caused by non-complicated URTI

Day from the start of treatment	Group 1: Homeopathic syrup alone (n = 46)	Group 2: Homeopathic syrup plus antibiotic treatment (n = 39)	*P* value^a^
2	−0.52 ± 0.66	−0.56 ± 0.55	0.673
3	−1.02 ± 0.75	−0.97 ± 0.74	0.830
4	−1.50 ± 0.84	−1.51 ± 0.68	0.833
5	−1.93 ± 0.88	−1.92 ± 0.96	0.881
6	−2.22 ± 0.99	−2.23 ± 1.01	0.814
7	−2.46 ± 0.98	−2.41 ± 1.29	0.839
8	−2.65 ± 0.87	−2.62 ± 1.09	0.837
9	−2.76 ± 0.87	−2.79 ± 1.03	0.850
10	−2.87 ± 0.88	−2.79 ± 1.00	0.723
11	−2.83 ± 1.00	−2.82 ± 1.00	0.888
12	−2.85 ± 0.99	−2.90 ± 0.94	0.880
13	−2.91 ± 0.96	−3.00 ± 0.92	0.826
14	−3.11 ± 0.99	−3.10 ± 0.99	0.945
15	−3.20 ± 1.02	−3.15 ± 1.01	0.842
16	−3.17 ± 1.10	−3.26 ± 0.99	0.725
17	−3.26 ± 1.08	−3.26 ± 0.94	0.911
18	−3.24 ± 1.06	−3.28 ± 0.86	0.911
19	−3.37 ± 1.02	−3.33 ± 0.90	0.739
20	−3.35 ± 1.04	−3.36 ± 0.90	0.993
21	−3.43 ± 1.00	−3.36 ± 0.90	0.737
22	−3.46 ± 1.00	−3.41 ± 0.91	0.867
23	−3.48 ± 1.03	−3.38 ± 0.96	0.649
24	−3.52 ± 0.98	−3.44 ± 0.94	0.675
25	−3.52 ± 0.98	−3.49 ± 0.91	0.866
26	−3.52 ± 0.98	−3.49 ± 0.91	0.866
27	−3.46 ± 1.00	−3.46 ± 0.94	0.930
28	−3.43 ± 1.03	−3.46 ± 0.94	0.867

The mean (± standard deviation) values of the reported changes in the verbal category-descriptive (VCD) scale *versus* the basal ones (Day 1) are shown. All changes in both groups were highly significant (*P* < 0.001; Wilcoxon matched-pairs signed-rank test)

^a^Kruskal-Wallis test

### Cough resolution

**Fig. 2:**
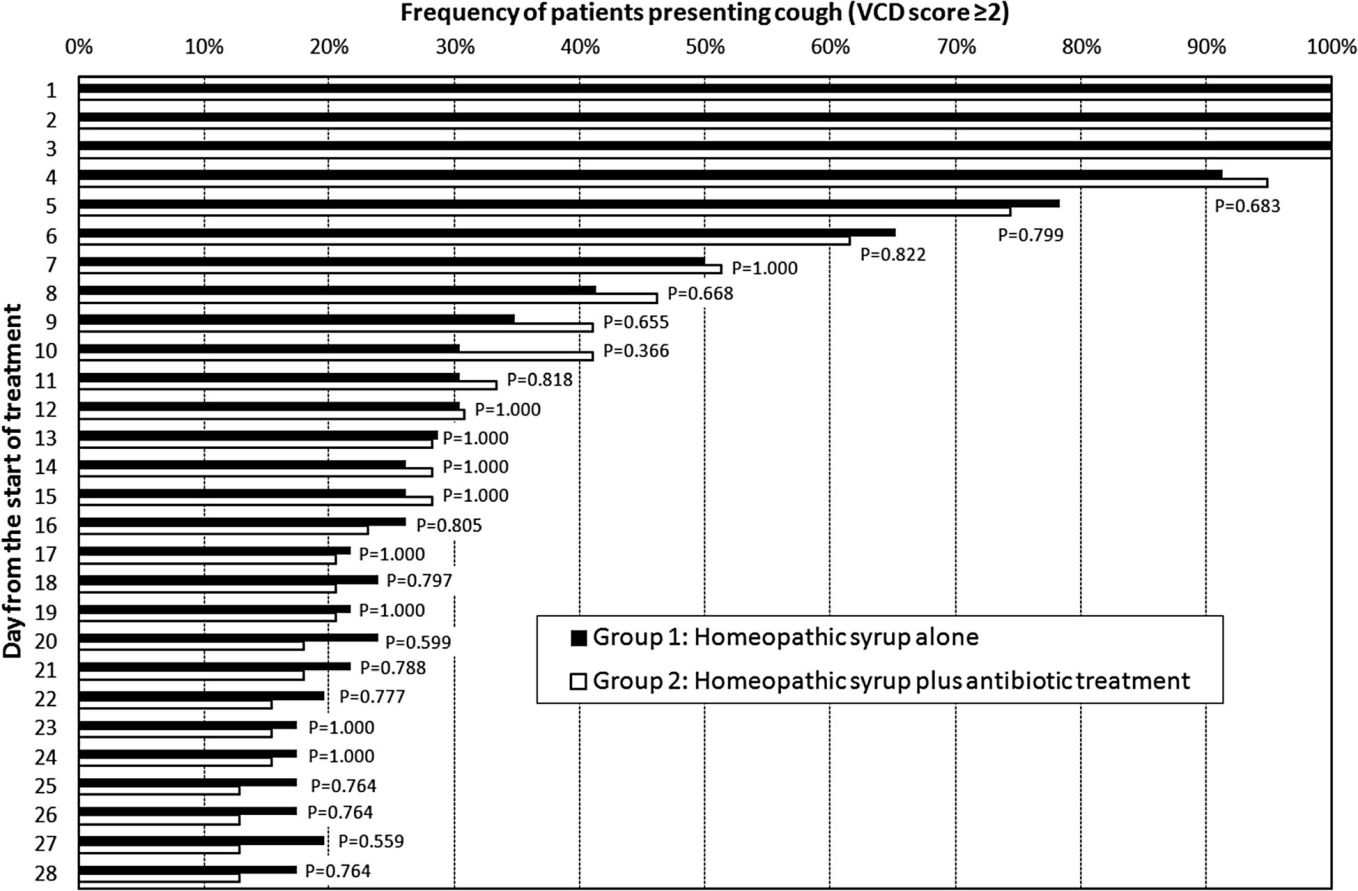
Cough resolution during the whole observational period in children affected by wet acute cough caused by non-complicated URTI. The Fisher’s exact test was applied

The analysis of patients presenting cough (i.e., VCD score greater than 1) on each day of the study is reported in Fig. 2. Cough resolution began on Day 4 (8.7 % in Group 1 and 5.1 % in Group 2) and continued progressively through the entire study period. In particular, after the first week (Day 8) cough was completely resolved in more than one-half of patients in both groups (58.7 % in Group 1 and 53.8 % in Group 2), while 22 % of patients in Group 1 and 17.9 % of patients in Group 2 were still coughing at Day 21. Cough was still reported in 8 patients of Group 1 (17.4 %) and in 5 patients of Group 2 (12.8 %) at the end of the observation, although the cough in those patients did not interfere with daily activities and sleep (i.e., VCD score equal to 2 or 3).

### Safety

We observed a total of 11 adverse events with a significantly (*P* = 0.020) higher frequency in patients who received syrup plus antibiotic treatment than in those who took syrup alone. In fact, two patients in Group 1 (4.3 %) reported insomnia (n = 1) and vomit (n = 1) while nine patients in Group 2 (23.1 %) reported diarrhea (n = 4), vomit (n = 3) and skin rash (n = 2).

## Discussion

Our group recently published a controlled randomized trial demonstrating the favorable effect of a homeopathic syrup on the resolution of acute cough in adults compared to placebo. [17]. Our data suggest an antitussive efficacy of this homeopathic syrup in children since the time-courses of the VCD severity score in the syrup treated children of this study resulted overlapping to that obtained in syrup treated adults in a randomized, double-blind placebo-controlled trail [17]. In fact, after 4 days the mean VCD score was a bit more than 2 both in children (present study) and in adults [17] *versus* a mean VDC score of more than 3 observed in adults treated with placebo [17].

The data of the present study also indicate that adding antimicrobial agents to the homeopathic syrup does not in any way benefit the symptomatic treatment--so adding to the weight of evidence against prescribing antibiotics to patients with acute cough due to uncomplicated URTI. The differences in VCD scale between the homeopathic syrup group and the group that also received antibiotics are not statistically significant on any observation day, and the cough resolution trend was comparable week after week for both groups (Table 2).

At the end of the second week of observation, cough was resolved in 74 % of children treated with syrup alone and in 72 % of children who received syrup plus antibiotic, while at the end of the 28-day observation period about 10–20 % of children still presented cough, without any significant difference between the two treatment groups. These data are consistent with a recent review on the duration of symptoms of respiratory tract infections in children [3]. The percentage of children still presenting cough at the end of the study confirms that acute cough associated with URTI continues for several weeks, thus suggesting that it is necessary to educate people and the medical community about this natural history.

For the assessment of cough severity we did not use a complete parent-compiled quality of life quality (PC-QoL) questionnaire but instead we adopted a subjective verbal category-descriptive (VCD) scale [18]. Although this scale was validated against an objective cough meter measure in children slightly older (6–17 year-old) than our population (4–15 year-old), it can be considered reliable also for our study since it is a parent-assisted card. The VCD was found to be easier to use than the PC-QoL and so assures better compliance; in fact, we have made a preliminary test on the correct compilation of the VCD scale and the PC-QoL questionnaire, conducted on 20 patients for 28 consecutive days, and we obtained a compliance of 95 % for VCD but just 35 % for PC-QoL. It should also be pointed out that VCD has been proven to have a high correlation with domain variations of the PC-QoL questionnaire in children [21, 22]. Furthermore, since the VCD scale was used in our previous study on the same homeopathic syrup in adults, applying the same validated assessment tool in this study on a child population enabled us to obtain standardized and comparable data between children and adults [17].

As far as safety is concerned, it is worth noting that a significant difference was found between the two groups of children: only two children in the group treated with syrup alone reported adverse effects, versus nine children in the group treated with the syrup plus antibiotics.

As far as the limitations of the research are concerned, a major weakness of our study arises from the observational design that we applied in this appraisal. Thus we did not considered a placebo group since the main goal of our research was not the effectiveness of homeopatic syrup but it was to evaluate the role of additional antibiotic. It should be pointed out that this research was a pilot study conducted before starting a larger trial on the role of antitussive and mucolytic drugs in children; however, the results of this preliminary study can provide valuable information for the sizing of future rigorous controlled studies, to be planned with a random allocation of patients to study groups.

## Conclusion

In conclusion, our data confirm that the studied homeopathic treatment has potential benefits on cough in children, as well as highlighting the good safety profile of this treatment. Supplementing the syrup with antibiotics did not improve cough resolution and was associated with more adverse events than the homeopathic syrup alone. These results indicate that antibiotics should not be routinely prescribed for uncomplicated acute cough secondary to URTI, as they are inappropriate for this condition and might be even dangerous-- leading to increased antimicrobial resistances and adverse events, without evidence of benefit [23–27].

## Abbreviations

PC-QoL: Parent-compiled quality of life
URTI: Upper respiratory tract infection
VCD: Verbal category-descriptive
